# Danish General Practitioners' Use of Prostate-Specific Antigen in Opportunistic Screening for Prostate Cancer: A Survey Comprising 174 GPs

**DOI:** 10.1155/2013/540707

**Published:** 2013-11-19

**Authors:** Kasper Jessen, Jens Søndergaard, Pia Veldt Larsen, Janus Laust Thomsen

**Affiliations:** Research Unit for General Practice, Institute of Public Health, University of Southern Denmark, J.B. Winsløws Vej 9A, 5000 Odense C, Denmark

## Abstract

*Background*. The use of prostate-specific antigen test has markedly increased in Danish general practice in the last decade. Despite the national guidelines advice against PSA screening, opportunistic screening is supposed to be the primary reason for this increased number of PSA tests performed. *Aims*. Based on the increase in the amount of PSA conducted, we aimed to analyse how GPs in Denmark use the PSA test. *Methods*. A self-administrated questionnaire concerning symptomatic and asymptomatic patient cases was developed based on the national and international guidelines and the extensive literature review, and an in-depth interview conducted with a GP was performed. *Results*. None of the GPs would do a PSA measurement for an asymptomatic 76-year-old man. For asymptomatic 55- and 42-year-old men, respectively, 21.9% and 18.6% of GPs would measure PSA. Patient request and concern could be potential reasons for measuring PSA for asymptomatic patients. Almost all GPs stated that a PSA measurement is indicated for symptomatic 49- and 78-year-old men, respectively, 98.9% and 93.8%. *Conclusion*. Opportunistic PC screening is being performed in general practice to a high degree. Hence, current guidelines are not followed, and intense focus should be on more effective implementation strategies in order to avoid overuse of PSA.

## 1. Introduction 

Prostate cancer (PC) in Denmark (DK) and many other western countries is the most prevalent cancer in men. In 2010, it caused 1210 deaths in Danish men, corresponding to a mortality rate of 20.4 per 100.000 men [1]. With 4060 new cases in 2010, it is still the most common cancer among men, with an incident rate of 142 per 100.000 [2]. Prostate-specific antigen (PSA) testing used as opportunistic screening could be a reason for the increase in incidence [3, 4]. In the Aarhus county in Denmark, the number of PSA measurements performed has increased 43-fold in the period from 1995 to 2006 [4]. Noteworthy, out of the total 86,077 included PSA samples, the proportion performed in general practice increased from 38.6% in 1998 to 66.1% in 2006 [4].

Two recent randomised controlled trials (RTC) found that PSA screening does not influence overall prostate cancer mortality. The European trial ERSPC found after a median follow up of 9 and 11 years a similar PC mortality rate ratio (RR) among men aged from 55 to 69, respectively, 0.80 and 0.79 [5, 6]. It is noteworthy to mention that only 2 of the 7 included centers in the ERSPC trial found a statistically significant reduction in PC mortality after 11 years and seemed to contribute to the overall benefit [6, 7]. Furthermore, no all-cause mortality reduction was found [6, 7], and PSA screening was associated with a significant overdiagnosis of 50% [8]—an adverse effect which is more common than screening for breast, colorectal, or cervical cancer [5, 9]. The American trial PLCO likewise has not found a mortality reduction [10]. 

A population-based cohort study has, however, found that the cumulative survival after PC in Denmark has increased in the period between 1998 and 2009. The 1-year cumulative survival increased from 80% (1998–2000) to 90% (2007–2009), and the expected increase in 3- and 5-year survival was 47%–73% and 34–60%, respectively [11]. The median age at PC diagnosis decreased from 74 in 1998–2000 period to 70 years in 2007–2009 period [11]. Hence, Borre et al. concludes that the survival results probably reflect patients being diagnosed earlier and therefore live longer with a cancer diagnosis then previously (lead-time bias) and patients with a slow growing cancer, with a better prognosis, will be diagnosed (length-time bias) rather then real improvement in survival [11].

Based on the current knowledge of adverse effects of PSA screening, neither opportunistic nor organised screening for PC is recommended [12], and there is a need for further insight into the mechanism behind the increased use of PSA in general practice. Hence, we aimed to analyse how GPs in Denmark use the PSA test.

## 2. Material and Methods 

We conducted a postal survey comprising 174 registered general practitioners (GPs) in Odense municipality (191.400 citizens), Denmark.

### 2.1. Setting and Sampling

All 174 registered GPs in Odense municipality (September 2010), Denmark, were invited to participate in the study. Some 98% of Danes are registered with GPs and receive free tax-financed medical care. Danish GPs are responsible for the frontline care, 24 hours a day, and act as gatekeepers for specialist and hospital referrals [13]. 

### 2.2. The Questionnaire

The self-administrated questionnaire with the case stories was developed based on a semistructured in-depth interview with one GP, on the literature on the use of PSA, and on the authors' clinical experience [5, 14]. Two of the authors are GPs (Janus Laust Thomsen and Jens Søndergaard). The questionnaire was pilot tested among GPs at the research unit department, and subsequent minor modifications were made. The final questionnaire comprised 15 items, corresponding to three case stories with patients not presenting symptoms of PC (asymptomatic) and two case stories with patients having potential PC symptoms (symptomatic). For each patient case, the respondents were asked whether or not they would measure PSA and about the rationale behind their decision. The asymptomatic case stories explore GP views on opportunistic screening, where age was suspected to be a significant factor for the relevance of a PSA measurement. Furthermore, the questionnaire explored GPs' views on the indication for measuring PSA. One of the factors included patients' concern and request. Another factor was the age interval of 55–69 where a mortality reduction has been demonstrated in the ERSPC trial [5]. It has been suggested that when an asymptomatic patient requests a PSA test, he should be informed about the possible consequences of a positive test [12]. This aspect was therefore also explored in the questionnaire. The symptomatic case stories explored whether LUTS and back pain are viewed as indicators for a test. The ages of 49 and 78 were chosen to explore whether age is considered a significant variable, regardless of patient symptoms. Aspects, such as family history and DRE findings, were also explored as potential indicators. The previous cases are illustrated in Table 2.

Together with each questionnaire, a postage paid envelope and a letter with information on the study were enclosed. Contact information was provided, and the GPs were encouraged to contact the authors if they had further questions. No reminder was sent. The GPs' demographic data was obtained from the National Board of Health through the authorisation register.

### 2.3. Statistical Analysis

With STATA software, the Fischer's exact test was used to assess the association between (i) the PSA indication and (ii) the rationale behind the indication. For some of the descriptive data, SPSS software was used. *P* values less than 0.05 were considered statistically significant.

### 2.4. Ethical Considerations

Each questionnaire was assigned numerical codes to ensure confidentiality, and the study was approved by the Danish Data Protection Agency.

## 3. Results

### 3.1. Participants/Descriptive Data

We sent questionnaires to 174 GPs. Due to postal circumstances (GP having moved, unknown on the address, or inadequate address) ten questionnaires were returned by the postal service and therefore not received by the GPs. Of the 164 eligible GPs, 98 returned the questionnaire yielding an overall response rate of 60%. The GPs demographic data can be seen in Table 1.

### 3.2. The Indication for Measuring PSA

With regard to an asymptomatic 76-year-old man, none of the respondents stated an indication for measuring PSA, neither if the patient was over 70 years old or if he had a life expectancy of at least 10 years. For an asymptomatic 55- and 42-year-old men, respectively, 21 (21.9%) and 18 (18.6%) of responders stated an indication for measuring PSA. It is, however, important to notice that for asymptomatic 55- and 42-year-old men, respectively, 22 (22.9%) and 14 (14.4%) of the responders did not know whether or not they would measure PSA. A patient's request and age interval of 55–69 were for 17 (81.0%) responders an indication for a PSA measurement for a 55-year-old man (*P* < 0.001). A patient's request and concern were for 17 (94.4%) responders an indication for a PSA measurement for a 42-year-old man (*P* < 0.001). All responders who stated an indication meant that a patient should have the opportunity for a PSA measurement if thoroughly informed about screening advantages and disadvantages (*P* < 0.001). Tables 2 and 3 illustrate the previous findings.

Almost all, 93 (98.9%), responders stated an indication for measuring PSA for a symptomatic 49-year-old man. Only one responder (1.1%) would not measure PSA. The indication was for 88 (94.6%) responders LUTS (*P* = 0.064) and for 73 (79.3%) responders family history (*P* = 0.215). Ninety (93.8%) responders state an indication for measuring PSA for a 78-year-old man, where LUTS and lower back pain were the indication for 89 (98.9%) responders (*P* < 0.001). Only two responders (2.1%) would not measure PSA and four responders (4.2%) did not know whether or not to measure PSA. The rectal examination finding does for 77 (88.5%) of the responders not have to be enlarged, hard and knobbly for it to be an indication for measuring PSA (*P* < 0.001). Tables 2 and 4 illustrate the previous findings.

## 4. Discussion

### 4.1. Primary Findings

Our primary finding was that a substantial proportion (one-fourth) of the GPs uses PSA as an opportunistic screening tool despite the national and international guidelines [7, 12]. The debate on whether to use PSA as early detection of PC is ongoing, and there is no international agreement in the approach to PSA screening. Recent international guidelines published this year have slightly different approaches to the current evidence on PSA screening. For example, The American Urological Association (AUA) does not recommend routine PSA screening in men aged 40 to 54 years old who are not at increased risk for the disease based on family history and race [15]. However, the European Association of Urology (EAU) has recently changed its recommendations from mentioning the age of 40 as a potential age on which screening intervals may be based [16] to now the recommendation that all men from 40 to 45 years of age should be offered a PSA measurement in order to initiate a risk adapted follow-up approach [17]. 

 It is important to keep in mind that the ongoing international debate and research might not get carried across to the ordinary GPs who may rely primarily on national guidelines advising against PSA screening.

### 4.2. Strength and Weaknesses

The response rate is high in comparison with other questionnaires to GPs (60%) [18, 19], and there is no indication that the responses were related to the use of PSA testing, and we have therefore no reason to believe that we have overestimated inappropriately the use of PSA in general practice. It is considered a point of strength that few missing data (2.0% to 5.1%) were found amongst responders (Tables 3 and 4). As there were no validated questionnaires on the topic, we developed a questionnaire, but future studies should be on further validation of similar questionnaires addressing GPs' use of tests with a screening potential. 

### 4.3. The Concern for Measuring PSA for Asymptomatic Patients

The GPs clinical practice regarding the use of PSA for asymptomatic patients is a concern since it is used against evidence-based national and international guidelines. The practice is a concern due to the lack of evidence on whether PSA screening results in mortality reduction [5, 6, 10] and whether the benefits outweigh the adverse effects. Due to PSAs limited sensitivity and specificity of 71.9% and 90%, respectively [20], it may lead to an unnecessary PC diagnosis, unnecessary investigations, and treatment that can be associated with adverse physical (erectile dysfunction and urinary incontinence [21]) and psychological effects [22].

### 4.4. Predictors for Ordering PSA

Potential factors and predictors which could influence the GPs decisional behaviour and prompt the GPs to measure PSA against guidelines could be a patient's *request* and *concern* [12]. The discomfort with uncertainty and the regret of overseeing a potential cancer probably increase if the patient is young and a previous request has been made [23, 24]. This could be why the GPs in this study measure PSA for young asymptomatic patients upon request, which also has been shown amongst GPs (60%) across the United Kingdom for men under the age of 55 [25]. This, and the fact that only 10% measure PSA due to the mortality reduction, can question the clinical practice since this is an essential purpose of screening.

Furthermore, it has been found that GPs are more likely to order a PSA measurement if the GP previously has diagnosed PC in an asymptomatic patient [26, 27] and if the GP would test himself [27]. Unfortunately, PSA screening is expected to increase due to media campaigns and pressure from patients [12, 28]. It, therefore, seems that the GP's own clinical experience and the pressure from patients overrule the recommendations in guidelines.

Some GPs “do not know” whether or not to measure PSA with regard to asymptomatic 55- and 42-year old men. This uncertainty could have an influence on how the GPs use the PSA test in their clinical practice. However, it is not possible from this study to suggest whether it will lead to an increase or decrease in the amount of PSA measurements performed.

Shared decision making is recommended as a way to overcome the problems of the uncertainties of PSA screening [15, 29]. However, the individual patient might not understand the meaning of discovering indolent cancers, overdiagnosis, and overtreatment [30], and since the information is influenced by the GPs opinion, the information given might be biased. Since the decisional behaviour is influenced by the GPs own opinions [31], and other factors as mentioned, this approach and the effectiveness of current guidelines could be questioned.

## 5. Conclusion

The general purpose of cancer screening is mortality reduction and the increase in life quality. Current national and international recommendations advice against PSA screening due to the lack of secure evidence of the benefits of PSA screening and the possible adverse effects with regard to screening. In contrast, opportunistic prostate cancer screening is often being offered in general practice. Hence, current guidelines are not followed, and intense focus should be on more effective implementation strategies in order to avoid overuse of PSA. 

## Figures and Tables

**Table 1:** Characteristics of responding GPs.

Demographic characteristic	Number (%)
Gender	
Male	66 (69)
Female	30 (31)
Age (years)	
≤50	30 (32)
≥51	64 (68)
Time in general practice (years)	
≤10	27 (29)
≥11	67 (71)

Missing data are excluded.

**Table 2:** 

GPs response to whether or not they would measure PSA.	PSA indication, *n* (%)		
	Yes	No	Do not know
*Case A *			
A 76-year-old man addresses his general practitioner for a general health checkup. He is healthy and has never been seriously ill. There are no changes in urinary symptoms and no family history of any illnesses.	0 (0.0)	96 (99.0)	1 (1.0)

*Case B *			
A 55-year-old man walks into your general practice for an annual health checkup. He is healthy and physically active, has normal weight, and has never been seriously ill. He wants to have taken blood tests for “everything,” including blood glucose, PSA, cholesterol, and lever function tests. There is no family history of any illnesses.	21 (21.9)	53 (55.2)	22 (22.9)

*Case C *			
A worried 42-year-old man walks into your general practice. Due to the increased media coverage and since his neighbor just has been screened for prostate cancer, he has now become worried and wishes a PSA test. He is healthy, has no lower urinary symptoms, and has no family history of any illnesses.	18 (18.6)	65 (67.0)	14 (14.4)

*Case D *			
A 49-year-old man is worried because he has recognized that his urination is not as usual. He has to urinate more often and has to urinate at night. He feels more tired but is otherwise healthy. The prostate is not enlarged. His father was diagnosed with prostate cancer when he was 68 years old.	93 (98.9)	1 (1.1)	0 (0.0)

*Case E *			
A 78-year-old man walks into your general practice to measure PSA. His urinary stream has weakened and is slow to start. The patient reports having lower back pain. On the physical examination, the patient reports tenderness corresponding to L4 when you tap the area. The prostate is palpated moderately enlarged but firm, smooth, and elastic. There is no family history for any illnesses.	90 (93.8)	2 (2.1)	4 (4.2)

**Table 3:** Subtables comparing the GPs responses to whether they would measure PSA and their rationale behind PSA indication for asymptomatic patients.

GPs responses to the corresponding questions regarding their rationale behind the PSA indication.	PSA indication*, *n* (%)			*P* value
	Yes	No	Do not know	
*Case A *				
All patients over the age of 70 should have measured PSA.				
Yes	0 (0.0)	1 (1.1)	0 (0.0)	1.000
No	0 (0.0)	90 (94.7)	1 (100.0)	
Do not know	0 (0.0)	4 (4.2)	0 (0.0)	
Patients with a life expectancy over 10 years should have measured PSA.				
Yes	0 (0.0)	1 (1.1)	1 (100.0)	0.021
No	0 (0.0)	84 (89.4)	0 (0.0)	
Do not know	0 (0.0)	9 (9.6)	0 (0.0)	

*Case B *				
A PSA measurement should be performed to diagnose an early stage PC, hence reducing mortality.				
Yes	2 (10.0)	2 (3.8)	1 (4.8)	0.004
No	10 (50.0)	44 (83.0)	10 (47.6)	
Do not know	8 (40.0)	7 (13.2)	10 (47.6)	
All men in the age interval 55–69, requesting a PSA measurement, should have the opportunity.				
Yes	17 (81.0)	3 (5.7)	4 (19.0)	<0.001
No	2 (9.5)	39 (73.6)	1 (4.8)	
Do not know	2 (9.5)	11 (20.8)	16 (76.2)	

*Case C *				
Concerned patients requesting a PSA measurement to exclude PC should have the opportunity.				
Yes	17 (94.4)	3 (4.7)	1 (7.1)	<0.001
No	0 (0.0)	48 (75.0)	3 (21.4)	
Do not know	1 (5.6)	13 (20.3)	10 (71.4)	
A patient well informed of advantages and disadvantages concerning PC screening should have the opportunity.				
Yes	18 (100.0)	20 (31.3)	8 (57.1)	<0.001
No	0 (0.0)	27 (42.2)	0 (0.0)	
Do not know	0 (0.0)	17 (26.6)	6 (42.9)	

*The proportion of missing data was between 2.0% and 4.1%.

**Table 4:** Subtables comparing the GPs responses to whether they would measure PSA and their rationale behind PSA indication for symptomatic patients.

GPs responses to the corresponding questions regarding their rationale behind the PSA indication.	PSA indication*, *n* (%)			*P* value
	Yes	No	Do not know	
*Case D *				
Patients with LUTS should have measured PSA.				
Yes	88 (94.6)	0 (0.0)	0 (0.0)	0.064
No	4 (4.3)	1 (100.0)	0 (0.0)	
Do not know	1 (1.1)	0 (0.0)	0 (0.0)	
The patient should have measured PSA, due to a family history of PC.				
Yes	73 (79.3)	0 (0.0)	0 (0.0)	0.215
No	10 (10.9)	1 (100.0)	0 (0.0)	
Do not know	9 (9.8)	0 (0.0)	0 (0.0)	

*Case E *				
Patients with LUTS and lower back pain should have measured PSA.				
Yes	89 (98.9)	0 (0.0)	0 (0.0)	<0.001
No	0 (0.0)	1 (100.0)	1 (25.0)	
Do not know	1 (1.1)	0 (0.0)	3 (75.0)	
A PSA should only be measured if the prostate is palpated enlarged, hard, and knobbly.				
Yes	6 (6.9)	2 (100)	1 (25.0)	<0.001
No	77 (88.5)	0 (0.0)	1 (25.0)	
Do not know	4 (4.6)	0 (0.0)	2 (50.0)	

*The proportion of missing data was between 3.1% and 5.1%.
